# A Comprehensive Mechanistic Antibacterial and Antibiofilm Study of Potential Bioactive ((BpA)_2_bp)Cu/Zn Complexes via Bactericidal Mechanisms against *Escherichia coli*

**DOI:** 10.3390/molecules28052215

**Published:** 2023-02-27

**Authors:** Sajid Ali, Nazma Faqir, Falak Naz, Muhammad Ishtiaq Jan, Naeem Khan, Amal Alotaibi, Riaz Ullah

**Affiliations:** 1 Department of Chemistry, Bacha Khan University, Charsadda 24420, Khyber Pakhtunkhwa, Pakistan; 2 Department of Chemistry, Kohat University of Science and Technology, Kohat 26000, Khyber Pakhtunkhwa, Pakistan; 3 Department of Basic Science, College of Medicine, Princess Nourah bint Abdulrahman University, P.O. Box 84428, Riyadh 11671, Saudi Arabia; 4 Medicinal Aromatic and Poisonous Plants Research Center, Department of Pharmacognosy, College of Pharmacy, King Saud University, Riyadh 11451, Saudi Arabia

**Keywords:** biphenylacetic acid (BpA), bipyridine (bp), biofilm, bactericidal, *Escherichia coli*, cell membrane, inhibitory potential, medical implants

## Abstract

Bacterial resistance to antibiotics and host defense systems is primarily due to bacterial biofilm formation in antibiotic therapy. In the present study, two complexes, bis (biphenyl acetate) bipyridine Cu (II) (**1**) and bis (biphenyl acetate) bipyridine Zn (II) (**2**), were tested for their ability to prevent biofilm formation. The minimum inhibitory concentration and minimum bactericidal concentration of complexes **1** and **2** were 46.87 ± 1.822 and 93.75 ± 1.345 and 47.87 ± 1.345 and 94.85 ± 1.466 μg/mL, respectively. The significant activity of both complexes was attributed to the damage caused at the membrane level and was confirmed using an imaging technique. The biofilm inhibitory potential levels of complexes **1** and **2** were 95% and 71%, respectively, while the biofilm eradication potential levels were 95% and 35%, respectively, for both complexes. Both the complexes showed good interactions with the *E. coli* DNA. Thus, complexes **1** and **2** are good antibiofilm agents that exert their bactericidal actions possibly by disrupting the bacterial membrane and interacting with the bacterial DNA, which can act as a powerful agent to restrain the development of bacterial biofilm on therapeutic implants.

## 1. Introduction

The emergence of pathogenic species resistant to antibiotics is one of the biggest issues facing the healthcare system [1]. Concern exists for both Gram-positive and Gram-negative bacteria in the absence of new operative antibiotics. The rise of the species resistance to the common antibiotics makes Gram-negative bacteria the topmost present consideration [2]. In the treatment of infections, the continuous arrangement of antimicrobial drugs has led to the emergence of resistance among the different strains of microorganisms [3]. Nowadays, various species of bacteria have become resistant to many classes of antibiotics because of horizontal gene transfer or selective pressure. The degree of resistance observed among *Enterobacter*, *Pseudomonas aeruginosa*, *Escherichia coli*, *Klebsella Pneumoniae*, *Staphylococcus aureus*, and *Acinetobacter baumannii* species is very dangerous, as such bacteria are the familiar etiologies for frequently detected community and hospital-developed contagions [4].

It has been thoroughly confirmed that bacterial biofilms are extremely tolerant to antibiotics [5]. Biofilms are a group of sessile bacteria attached and rooted to a matrix surface. Biofilms prevent the penetration of antimicrobial agents, thereby interfering with the mechanisms of action of these agents, leading to persistent infections [6].

There are five stages of biofilm formation: (1) reversible attachment and planktonic movement; (2) permanent attachment as well as monolayer colony formation; (3) a flat micro-settlement pattern; (4) large colony formation with a mushroom-like structure; (5) dispersion and reattachment [7]. Attachment to a surface and colony formation are key factors that are primarily required for biofilm development [8]. *E. coli* requires motility to colonize in the host for infection [9].

Recently, organic ligands have been replaced by metal complexes in their biological activity. Thus, the biological efficacy is expected to be improved by chelating appropriately designed ligands with metal ions [10]. However, a variety of organic ligands and their derivatives have the significance of being used as antiamoeba, antihistamine, deworming, antiulcer, antifungal, anticancer, and antihypertensive drugs [11,12,13,14,15,16]. 

Copper is an important transition metal required for several biological functions in terms of Cu-enzymes and blue proteins, but also because of their potential synergistic activity towards drugs [17,18]. Copper (II) complexes with different organic ligands exhibit augmented activity compared to drugs because of their potential antitumor, antioxidant, antibacterial, and antifungal activities [19]. Recently, the antimicrobial applications have been studied. These complexes are used in oral hygiene products, fungicides, pesticides, antimicrobial drugs, medical health equipment, preservatives, and other applications [20]. Similarly, zinc compounds also possess antibacterial properties, such as zinc chloride, which cures halitosis and periodontal disease [21,22]. Previous studies have shown the antimicrobial properties of zinc compounds and transition metal complexes in damaged tissue with no or little side effects [23,24,25,26,27,28].

During the drug design process, the toxicity of the active molecule is an important factor, such as the hemolytic activity. General cytotoxicity toward normal healthy cells is indicated by any compound’s hemolytic activity [29]. The complexes used in the present study are novel and their chemical structural data have been previously reported [30]. The aim of the present research work is to find out the antibacterial potential and antibiofilm efficacy of copper and zinc complexes against *E. coli* and to determine the effects on the bacterial membrane and DNA.

## 2. Results and Discussion 

### 2.1. Antibacterial Applicability of the Complexes

**Figure 1:**
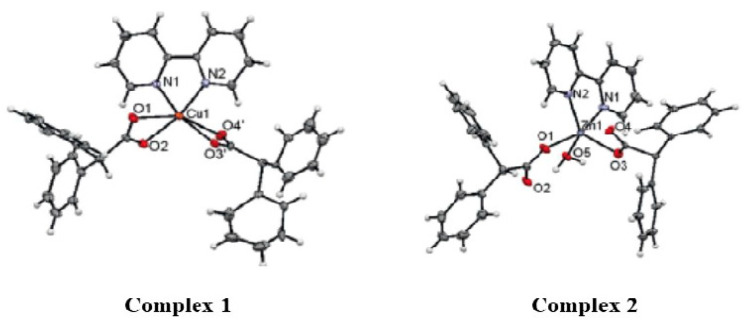
X-ray crystallographic structure of complex **1** and complex **2**.

**Figure 2:**
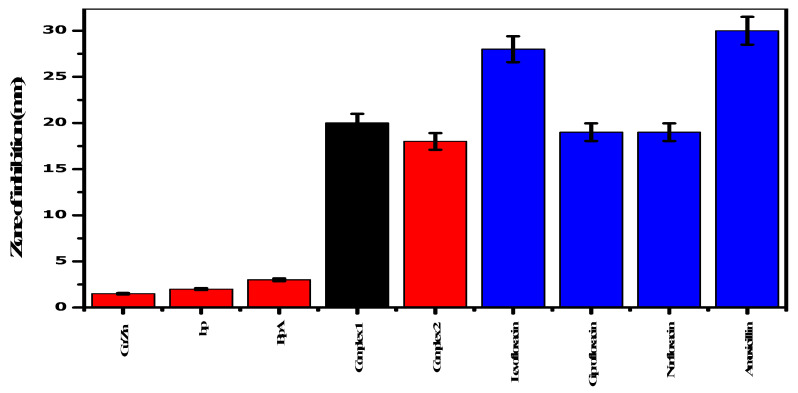
Antibacterial screening of complexes and commercial antibiotics for *E. coli.*

Both complexes (Figure 1) showed antibacterial activity compared with the standards, as shown in Figure 2. The zones of inhibition for complexes **1** and **2** measured 20 and 18 mm, respectively. Similarly, the minimum inhibitory concentration (MIC) values of the test complexes were evaluated using the dilution method. The MIC values for complexes **1** and **2** were 46.87 ± 1.822 and 47.87 ± 1.345 µg/mL, respectively.

Similarly, the minimum bactericidal concentration (MBC) of the test complexes was calculated from the tubes used for the MIC testing by transferring the culture via spot inoculation onto nutrient agar plates. The lowest concentrations of the test complexes that resulted in no visible growth of bacteria were considered as the MBC of the test complexes, which equaled 93.75 ± 1.345 and 94.85 ± 1.466 µg/mL for complex **1** and complex **2**, respectively (Table 1). 

### 2.2. Effects of the Complexes on Membrane Integrity 

**Figure 3:**
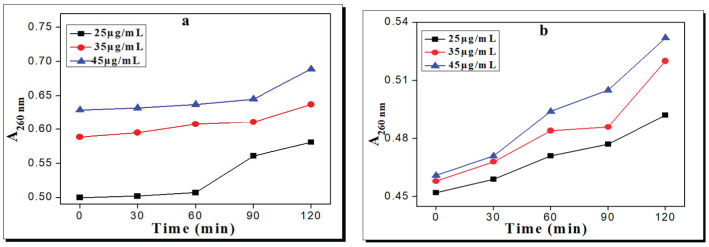
Release of extracellular materials absorbed at 260 nm from *E. coli* cell suspensions by (**a**) complex **1** and (**b**) complex **2** at different times and concentrations.

The treatments of *E. coli* with complex **1** and complex **2** showed marked increases in A_260_ values with increases in the concentration and exposure time, as these results revealed that both complexes caused serious damage to the bacterial membrane. The membrane disruption potential of complex **1** was increased as the concentration and time exposure were increased. The A_260_ values were 0.581, 0.637, and 0.689 at 25, 35, and 45 µg/mL for complex **1**, respectively. Similarly, in the case of complex **2**, the A_260_ values were 0.492, 0.52, and 0.532 at 25, 35, and 45 µg/mL, respectively. Thus, complex **1** displayed greater membrane disruption potential than complex **2** because of the better interaction of complex **1** with the bacterial membrane, as shown in Figure 3a,b. 

### 2.3. Effects of the Complexes on Inner Membrane Permeability

**Figure 4:**
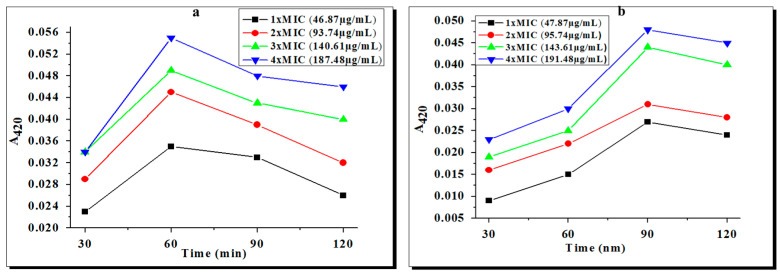
Release of cytoplasmic β-galactosidase from cell suspensions of *E. coli* treated with (**a**) complex **1** and (**b**) complex **2** at different times and concentrations.

This bioassay is based upon the β-galactosidase releases present in the inner membrane of *E. coli*. The actual substrate for β-galactosidase is lactose. This enzyme cleaves the mimic molecule (ONPG) into a chromogenic product o-nitrophenol, which was measured at 420 nm [31]. Bacterial cells were exposed to varying concentrations (i.e., 1 × MIC, 2 × MIC, 3 × MIC, and 4 × MIC) of zinc and copper complexes, and the cleavage rates were measured, as shown in Figure 4a,b. The maximal hydrolysis rate of ONPG was obtained at 60 min for complex **1**, while for complex **2** it was obtained at 90 min. After the reported time interval, the rate of ONPG hydrolysis was reduced, strongly suggesting a loss of enzyme activity. However, complex **1** exhibited severe inner-membrane-damaging activity with an increase in the dose, as shown by an enhanced A_420_ value, indicating greater permeability for its entry than complex **2**. The possible mechanisms for the bactericidal actions of these complexes might be the binding of the complexes to the membrane or the passive diffusion of complexes through the cell membrane, resulting in membrane permeability. 

### 2.4. Effects of the Complexes on Biofilm Eradication 

**Figure 5:**
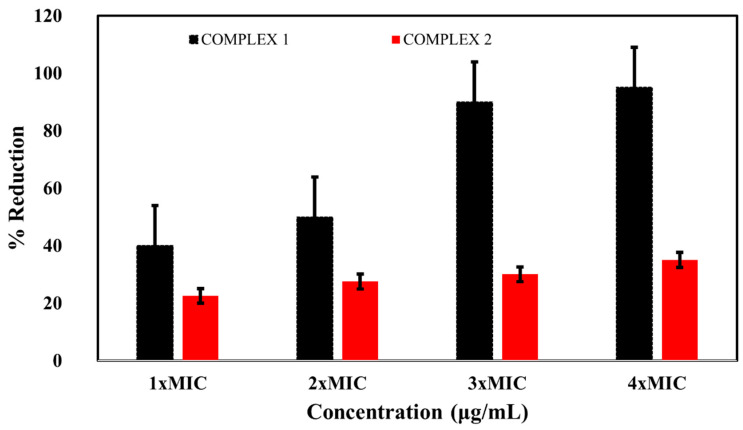
Eradication of *E. coli* biofilms at different concentrations of complexes.

The removal of biofilm-embedded bacteria was observed after the treatment of preformed biofilms with increasing concentrations of complex **1** and complex **2**. Complex **1** at 1 × MIC, 2 × MIC, 3 × MIC, and 4 × MIC exterminated 40, 50, 90, and 95% of the established biofilm, respectively. Similarly, complex **2** at 1 × MIC, 2 × MIC, 3 × MIC, and 4 × MIC eradicated 22.5, 27.5, 30, and 35% of the preformed biofilm, respectively (Figure 5). These results showed that both the complexes eradicated the established biofilm in a concentration-dependent manner; however, the biofilm eradication potential of complex **1** was greater than that of complex **2**. Thus, complex **1** at concentrations of 4 × MIC and higher showed potential for the control of biofilm formation. 

### 2.5. Biofilm Inhibition Activity 

**Figure 6:**
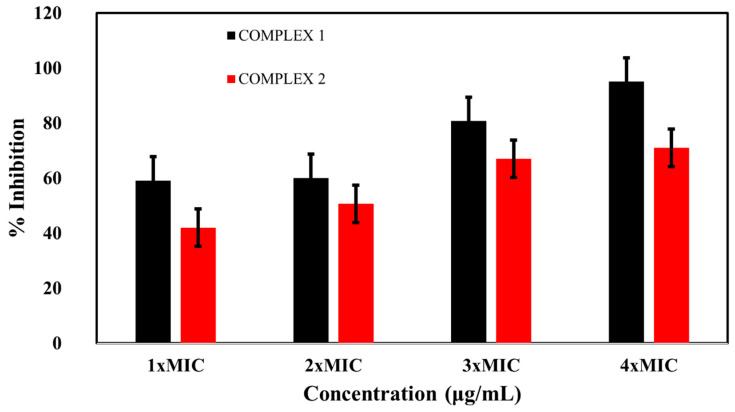
Percentages of inhibition of *E. coli* biofilm formation by complex **1** and complex **2**.

Both complexes showed greater inhibition of *E. coli* biofilm formation with increasing concentrations. Complex **1** caused 59, 60, 80, and 95% inhibition of the *E. coli* biofilm at 1 × MIC, 2 × MIC, 3 × MIC, and 4 × MIC, respectively. Similarly, the inhibition rates of the biofilm by complex **2** were 42, 50, 67, and 71% at the mentioned concentrations, respectively (Figure 6). These results suggest that both complexes have the ability to inhibit biofilm formation.

### 2.6. Effects of the Complexes on Bacterial DNA 

**Figure 7:**
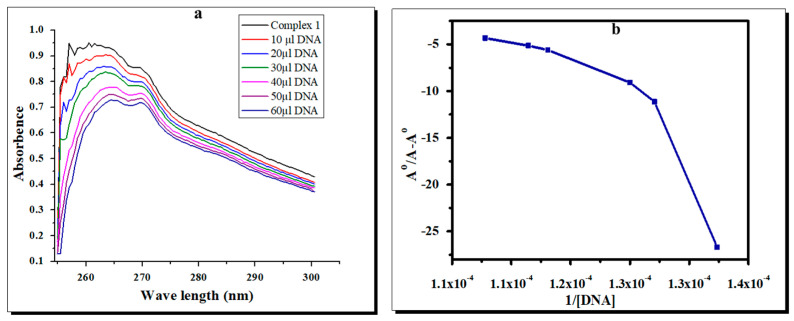
(**a**) UVVis spectra of the Cu complex with DNA at different concentrations of the complex and blank (**b**) A°/A−A° vs. 1/[DNA] and the binding constant for the Cu complex with *E. coli* DNA.

**Figure 8:**
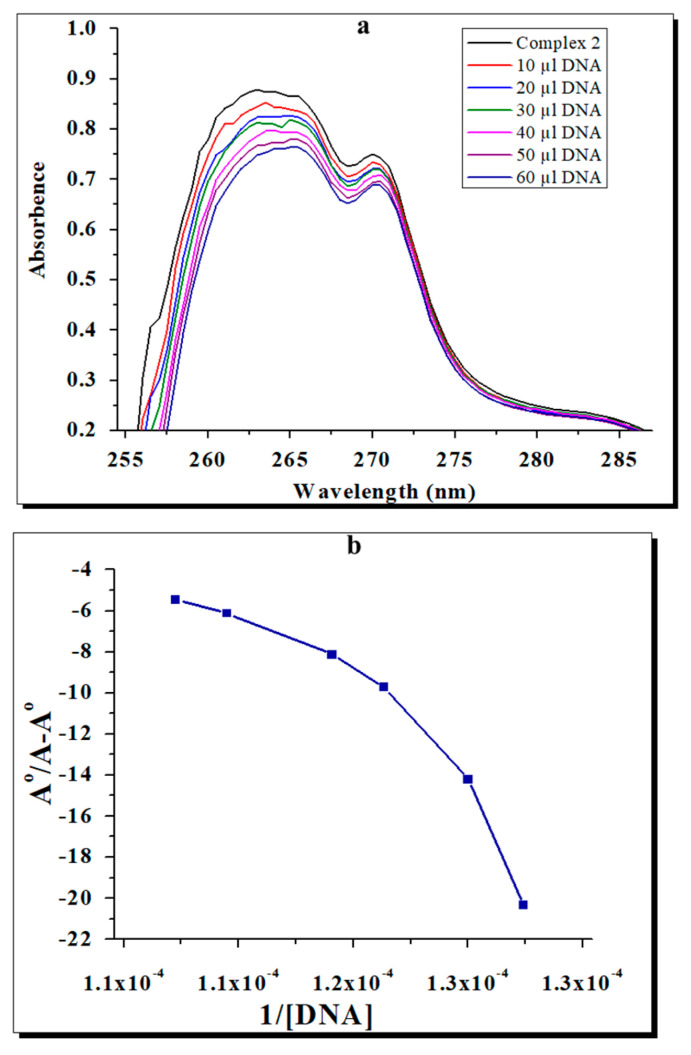
(**a**) UV-Vis spectra of the Zn complex with DNA at different concentrations of the complex and blank (**b**) A°/A−A° vs. 1/[DNA] and the binding constant for the Zn complex with *E. coli* DNA.

**Figure 9:**
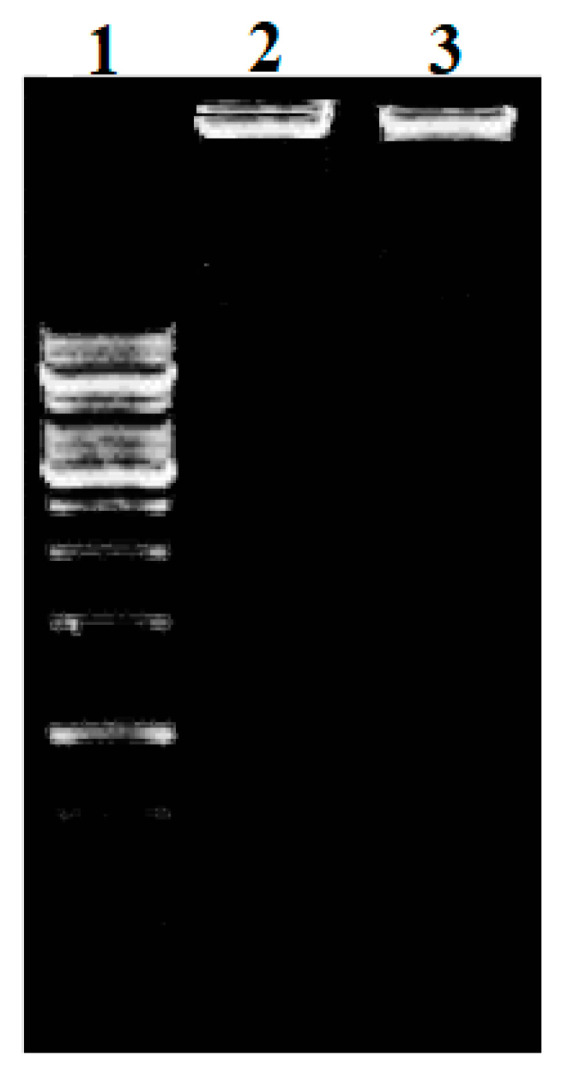
Patterns of *E. coli* DNA binding with test complexes: (**1**) untreated DNA (lane 1); (2) DNA treated at 4 × MIC of complex 1 (lane 2); (3) DNA treated at 4 × MIC of complex 2 (lane 3).

The copper and zinc complexes showed a hypochromic effect with the expansion of the DNA, demonstrating a reasonable association between DNA and the complexes. A red shift in wavelength of the absorption peak (λ max) was seen in both complexes. The absorption rates of complexes **1** and **2** in the presence and absence of DNA are displayed in Figure 7 and Figure 8. By using the Benesi–Hildebrand equation, the binding constant (Kb) values of the complexes can be calculated from the intercept-to-slope ratios of the Ao/(A-Ao) versus 1/[DNA] plots. The Kb values for complexes **1** and **2** were found to be 7.1 × 10^5^ M^−1^ and 7.5 × 10^5^ M^−1^, respectively [32,33,34,35]. The interaction of the test complexes with bacterial DNA was further confirmed through agarose gel electrophoresis (Figure 9). The test complexes at 4 × MIC displayed strong interactions with the bacterial DNA. In addition, compared with the control (lane 1), complex **1** (lane 2) and complex **2** (lane 3) caused more retardation in the mobility of the genomic DNA compared with the neat DNA, as the untreated DNA was lighter than the DNA chelated with the test complexes. Furthermore, both complexes do not cause the fragmentation of treated DNA and justify the bactericidal nature of the test complexes.

### 2.7. Electron Microscopic Analysis

**Figure 10:**
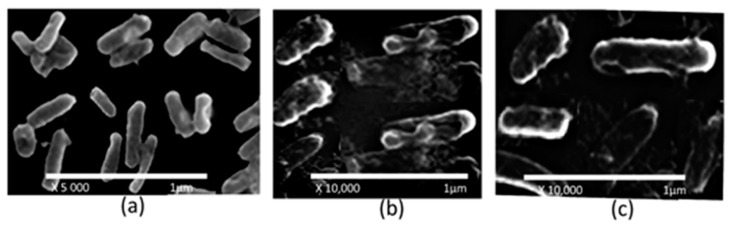
Scanning electron microscopy images of *E. coli*: (**a**) untreated culture; (**b**) *E. coli* culture treated with complex 1 (45 μg/mL); (**c**) *E. coli* culture treated with complex 2 (45 μg/mL).

The effect of the test complexes on the morphology of *E. coli* was studied using scanning electron microscopy (SEM). The cells treated with complex **1** at a concentration of 45 μg/mL showed drastic changes in morphology with respect to the cell wall. Similarly, complex 2 at the same concentration (45 μg/mL) caused alterations in the morphology of the bacterial cells, such as shrinking of the bacterial cells, which were probably due to damage to the cell wall (Figure 10). 

### 2.8. Hemolytic Activity

**Figure 11:**
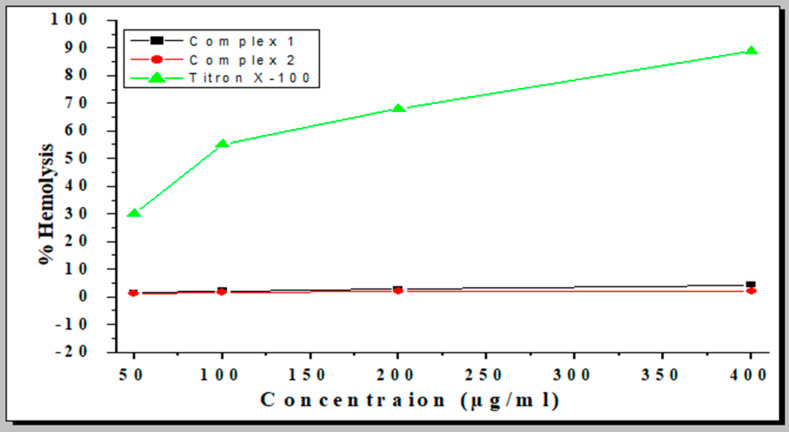
Percentages of hemolysis of red blood cells induced by complexes 1 and 2.

The test complexes’ hemolytic effects were measured at concentrations of 50, 100, 200, and 400 µg/mL, respectively. Complex **1** caused 1.3, 2.1, 2.8, and 4.2% lysis of the red blood cells at 50, 100, 200, and 400 µg/mL, respectively. Similarly, complex **2** caused 1.3, 1.6, 1.9, and 2% lysis of the red blood cells at 50, 100, 200, and 400 µg/mL, respectively. Triton-X, a standard hemolytic agent, exhibited 89% hemolysis at 400 µg/mL. These results showed that both complexes exhibited no hemolytic activity at 400 µg/mL, as shown in Figure 11. 

The main objectives of the present research work were to evaluate the antibacterial and antibiofilm potential of copper and zinc complexes of biphenyl acetic acid and bipyridine and to study the possible mechanisms behind the bactericidal actions of these complexes. Previous research work revealed that metal complexes of various ligands exhibited outstanding results [36]. Both in vitro and in vivo analyses showed that the development of biofilm by the bacteria makes them more resistant to antibiotics [5]. *E. coli* is resistant to several available antibiotics due to the thick lipopolysaccharide outer membrane. Biofilm formation by bacteria is associated with several bacterial infections, which finally lead to chronic diseases due to the low sensitivity of the bacteria to the available antibacterial agents [37]. *E. coli* is one of the biofilm-producing bacteria that secretes extracellular polymeric substances (EPS). These EPS are the characteristics of biofilms and play a major role in cell–cell or cell–surface attachment, acting as the initial protective barrier for bacteria and preventing the entrance of drugs [38]. The copper and zinc complexes of bis (biphenyl acetate) bipyridine exhibited significant antibacterial potential against *E*. *coli,* a Gram-negative pathogenic bacterium. The test complexes inhibited the biofilm formation in a concentration-dependent manner by preventing the adhesion of bacterial cells to the supporting surface. The MIC and MBC values of the complexes were significant and were comparable to standard antibiotics. Furthermore, these complexes also have the ability to eradicate the bacterial biofilm. This shows that the test complexes can be used as good candidates for the inhibition and eradication of biofilms by Gram-negative bacteria. The perturbation of the bacterial membrane by complexes **1** and **2** may occur via disrupting lipid–protein interactions and consequent increases in membrane permeability, which may cause further changes in the membrane structure and increased leakage of the intracellular constituents. This causes a resultant disruption of the membrane integrity and facilitates the entry of antibacterial agents [39]. The test complexes cause damage to the bacterial cell membrane, as evident from an increase in A_260_ values. Similarly, both the complexes also increase the permeability of the bacterial inner membrane, as shown by the overproduction of o-nitrophenol.

The UV-Vis spectrophotometry and agarose gel electrophoresis analysis showed that both complexes **1** and **2** have antibacterial activity levels by binding with bacterial DNA. In addition, the differences in λ max and absorbance values showed the interactions of complexes with bacterial DNA. Similarly, the gel electrophoresis study revealed that both complexes caused the condensation of the bacterial test DNA. After binding with bacterial DNA, the complexes interfered with cellular functions such as replication, transcription, and translation, which ultimately led to bacterial death. Thus, complexes **1** and **2** could be good therapeutic agents for managing bacterial infections without inducing resistance. 

## 3. Materials and Methods

### 3.1. Culture Collection 

The *Escherichia coli* (culture) was collected via pathology from HMC, Peshawar Pakistan. The nutrient agar medium, nutrient broth, and antibiotics were obtained from Merck, Germany. The ONPG (o-nitro phenyl-β-D-galactosidase) was purchase from Shanghai Malkin Biochemical Co., Ltd. (Shanghai, China). All other reagents and chemicals were purchased from Sigma Aldrich.

### 3.2. Synthesis of Bis (Biphenyl Acetate) Bipyridine Copper (II)

The bis (biphenyl acetate) bipyridine copper (II) was prepared by reacting biphenyl acetate with a copper sulphate (0.20 g, 1.25 mM) solution using continuous stirring for 3 h, followed by the addition of pyridine to the reaction at 60 °C. Complex **1** was obtained as a precipitated blue product, which was washed thoroughly with water and air-dried. 

### 3.3. Synthesis of Bis (Biphenyl Acetate) Bipyridine Zinc (II)

The bis (biphenyl acetate) bipyridine zinc (II) was prepared by treating biphenyl acetate with the solution of ZnCl_2_ with constant stirring for 3 h. Then, pyridine was added to the reaction mixture and allowed to stir for 3 h. Complex **2** was obtained as a white precipitate, which was washed thoroughly with water and recrystallized. 

### 3.4. Culture Conditions and Antibacterial Assay

A culture of *E. coli* was prepared by suspending a freeze-dried powder of *E. coli* in broth medium and kept at 37 °C for 24 h in a shaking incubator. The culture was aseptically streaked on an agar plate and allowed to grow at 37 °C for 24 h. The plate was stored in the refrigerator for further use. The antibacterial activity levels of complexes **1** and **2** were determined using the agar well diffusion method. The culture of the test bacterium was prepared according to the instructions of NCCLS. The test bacterium was incubated for 3–4 h in broth medium on a shaking incubator to attain a culture with 0.5 turbidity in McFarland units. The freshly prepared inoculum of the test bacterium was uniformly spread over the agar medium and allowed to stand for 3–5 min. Finally, with the help of a sterilized cork borer, wells were bored and 1 mg/mL samples of test compounds were added, followed by incubation at 37 °C for one day. The zones inhibited by the test samples were measured in mm, and the results were compared with standard antibiotics [40].

The broth dilution method was used to determine the minimum inhibitory concentration (MIC) of the test compounds with slight modifications [41]. Autoclaved broth medium samples were placed in a series of tubes and seeded with the bacterial test inoculum. The test compounds were added to the first tube at a concentration of 400 μg/mL and were diluted serial-wise to 12.5 μg/mL. Finally, all tubes were kept at 37 °C for 24 h and examined for bacterial growth. The lowest concentration at which the bacterial growth was inhibited was considered the MIC for the respective compound. The minimum bactericidal concentration (MBC) values of the test compounds were determined by transferring the sample from MIC tubes onto the agar medium plates, followed by incubation for 24 h. The lowest concentration that produced no growth of the test bacteria on the agar plate was considered the MBC of the test compounds. 

### 3.5. Cell Membrane Spoil Assay

The potential of the test complexes (**1** and **2**) for outer membrane spoilage was assessed by determining intracellular substances that tear upon interaction with bacterial cells [42]. The test bacterium from the agar plate was transferred and incubated at 37 °C for 24 h in the broth medium. The one-day-old culture was centrifuged and suspended in PBS solution. Finally, the optical density of the resulting culture was calibrated to 0.7 at 420 nm. Then, the different concentrations of test samples (1.5 mL) were mixed with the bacterial inoculum (1.5 mL), and the released intracellular substances at different time intervals were measured using a UV-Vis spectrophotometer.

### 3.6. Inner Cell Membrane Spoil Assay

The released β-galactosidase enzyme was measured using the reported procedure for the determination of the integrity of the inner membrane [43]. The bacterial inoculum was prepared using log-phase bacteria in 2% lactose nutrient broth medium. The test bacterium was harvested by centrifugation and suspended again in PBS, and the optical density was adjusted to 1.2 at 420 nm. Different concentrations of the test sample were added to the bacterial inoculum with the simultaneous addition of 150 µL of 30 Mm ONPG. The products of the reaction (*o*-nitrophenol) at different time intervals were measured at 420 nm.

### 3.7. Biofilm Eradication Assay

The crystal violet assay was used to measure the complexes’ ability to eradicate the biofilm with slight modifications [44]. The bacterial culture was grown overnight at 37 °C for 24 h on broth media. In addition, for an OD_600_ of 0.1, the culture was diluted to 1:50 in NB. Next, 1.8 mL of broth was mixed with 200 μL of diluted culture and the bacteria in broth media were given 24 h to produce biofilms. The negative control was chosen to be an equal volume of media. The complexes were then put in appropriate glass tubes at various concentrations and incubated for 24 h at 37 °C. After cleaning the dried tubes and aspirating the tubes’ contents, they were dyed with 0.1% crystal violet dye following the addition of 2 mL of 30% acetic acid to each tube. The biomass was then measured based on the optical density (OD) at 570 nm after 5–10 min of incubation at room temperature. The assay was performed in triplicate, and the results were compared with the control.

### 3.8. Biofilm Inhibition Assay

The culture of the test bacterium was prepared in nutrient broth media up to 5 × 10^5^ CFU/mL in freshly prepared nutrient broth with 2% glucose. Then, 90 µL of the diluted bacterial culture was added to the test complexes at concentrations of 1 × MIC, 2 × MIC, 3 × MIC, and 4 × MIC to get a final volume of 2 mL and incubated at 37 °C for 24 h. After the completion of the incubation time, the media was aspirated out and the bacteria biofilm was fixed with 100% methanol and air-dried. The staining was performed with crystal violet (CV) dye for 30 min. Finally, the tubes were washed with water and 2 mL of acetic acid (30%) was added to each tube. The absorbance was measured at 595 nm.

### 3.9. DNA Isolation and Binding

The bacterial DNA of selected species was extracted using a Gene JET Genomic DNA Purification Kit (thermos scientific) following the procedure provided with kit. The DNA was dissolved in distilled water to create a stock solution of the isolated bacterial DNA. The molar absorption coefficient at 6600 M^−1^ cm^−1^ was used to calculate the concentration of the stock DNA solution using a UV spectrophotometer at 260 nm. The complexes were prepared as aqueous DMSO solutions. The concentration of complexes was kept constant while the amount of DNA added was gradually increased in the range of 10–90 M. The solutions were incubated for 20–30 min at room temperature and the results were recorded using a UV–visible spectrophotometer. The cleavage of the bacterial DNA was checked through agarose gel electrophoresis. DNA samples treated with test complexes **1** and **2** were mixed with bromophenol blue and loaded. The untreated DNA sample was used as a control. Electrophoresis of the test samples was performed for 1 h, and finally the stained gel was illuminated below UV light and recognized.

### 3.10. SEM Analysis

The cell morphology and biofilm architecture were determined through a scanning electron microscopy analysis after studying the effects of the complexes on the shrinkage of the bacterial cells [45]. In addition, investigating the cell morphology, the test bacterium was incubated with or without test complexes 1 and 2 for 2 h. At the end of the incubation period, the bacteria were harvested by centrifugation at 4000 rpm. The bacterial pellet obtained was suspended again in PBS, after preparing a cover over the slide, and then fixed with glutaraldehyde for 2 h. The bacterial smear was dried with increasing concentrations of ethanol from 50 to 100% followed by liquid CO_2_. Finally, in a sputter coater, the bacterial sample was coated with gold and the sample SEM photographs were observed. Tissue culture plates with a glass coverslip were used for the formation of biofilms. The optical density of the test bacterium was adjusted to an absorbance level of 0.1 at 600 nm. Sterilized nutrient broth medium and varying concentrations of the test complexes were added to the test wells containing tissue culture plates and inoculated with the test inoculum. After incubation for a period of 24 h under static conditions at 37 °C, the desired medium and the wells were washed thrice with PBS (0.01 mol/L). The biofilm formed on the coverslip was fixed with glutaraldehyde, washed twice with PBS, and dried with ethanol at concentrations ranging from 50–100%. The dried glass coverslips were sputtered and examined under a scanning electron microscope.

### 3.11. Hemolytic Assay 

Human blood samples were collected in K_2_-EDTA-coated tubes to prevent blood coagulation. The plasma samples were aspirated gently after blood centrifugation for 5 min at 14,000 rpm. In addition, PBS (pH 7.4) was used to rinse the blood cells. The blood cells were diluted with different concentrations of complexes at a 1:1 ratio. Both negative (PBS) and positive (Triton −100 (20%)) controls were run for comparison. A Shaker incubator was used to incubate the tubes at 37 °C for 1 h. The content was subjected to centrifugation for 5 min at 14,000 rpm. The absorbance of the supernatant was assessed at 540 nm. The percentage hemolysis was calculated with the following formula:

$$\%\;Hemolysis=\;\frac{At-An}{Ap-An}*100$$

where the absorbance of the tested sample “*t*” is represented by *At*, the absorbance of the negative control is represented by *An*, and the absorbance of the positive control is represented by *Ap* (Triton-X-100).

### 3.12. Statistical Analysis

SPSS 21 (IBM Corp) was used for the analysis of statistically significant values among different groups followed by a one-way ANOVA with Tukey’s post hoc test. Here, *p <* 0.05 was considered significant. The data are expressed as means ± SD.

## 4. Conclusions

In the present study, two complexes, i.e., complex **1** (bis (biphenyl acetate) bipyridine copper (II)) and complex **2** (bis (biphenyl acetate) bipyridine zinc (II)), were evaluated for their antibacterial and antibiofilm activities and their possible bactericidal mechanisms were determined. The significant minimum inhibitory concentration and minimum bactericidal concentration values showed potential antibacterial activity for both complexes. The bacterial membrane disruptive potentials of the test complexes were substantial. However, the potential of complex **1** was more important than complex **2**. Both complexes caused enhanced permeation of the inner membrane. Both complexes had a greater capacity to hinder the formation of bacterial biofilms while eradicating the capability of the existing biofilm. Both the complexes were permeable to the bacteria and bound with the bacterial DNA, resulting in its cleavage, which consequently altered cellular functions such as replication, translation, and protein synthesis, which ultimately led to the death of the bacterium. Scanning electron microscopy studies revealed that both complexes caused drastic changes in the morphology of bacterial cells, such as shrinkage of the bacterial cells, which might have been due to the damaged caused to the cell walls. This study confirmed that the test compounds are capable of killing *E. coli*, meaning they can be employed as potent antibacterial and antibiofilm agents for the prevention of pathogenesis caused by *E. coli*.

## Figures and Tables

**Table 1:** Minimum inhibitory concentration (MIC) and minimum bactericidal concentration (MBC) values of complexes and commercial antibiotics for *Escherichia coli.*

S.NO	Sample	MIC (μg/mL)	MBC (μg/mL)
1	Complex 1	46.87 ± 1.822	93.75 ± 1.345
2	Complex 2	47.87 ± 1.345	94.85 ± 1.466
3	Levofloxacin	11.71 ± 1.334	23.43 ± 1.345
4	Ciprofloxacin	46.77 ± 1.456	98.75 ± 1.765
5	Norfloxacin	48.87 ± 1.765	99.15 ± 1.433
6	Amoxicillin	49.97 ± 1.623	99.75 ± 1.222

## Data Availability

All the available data incorporated in the MS and can be found with Sajid Ali.

## Sample Availability

Samples of the compounds are available and provided by first author Sajid Ali (Department of Chemistry, Bacha Khan University Charsadda Pakistan).
